# Supplementary material for: The Evolution and Characterization of the RNA Interference Pathways in Lophotrochozoa
Source: Genome Biol Evol. 2024 May 7;16(5):evae098. doi: 10.1093/gbe/evae098 (PMC11114477; doi:10.1093/gbe/evae098)
Supplement: evae098_Supplementary_Data [file evae098_supplementary_data.zip › Supplementary_materials.zip!/selected_miRNAs/DaRe_selected_miRNas/DaRe_NW_018394988.1_46253.pdf]

Provisional ID	:	NW_018394988.1_46253
Score total	:	98.5
Score for star read(s)	:	3.9
Score for read counts	:	90.4
Score for mfe	:	3.3
Score for randfold	:	1.6
Score for cons. seed	:	-0.6
Total read count	:	189
Mature read count	:	151
Loop read count	:	0
Star read count	:	38

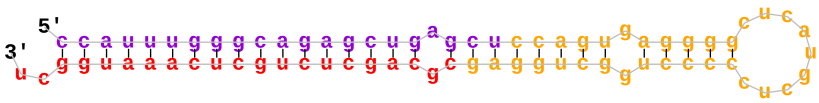

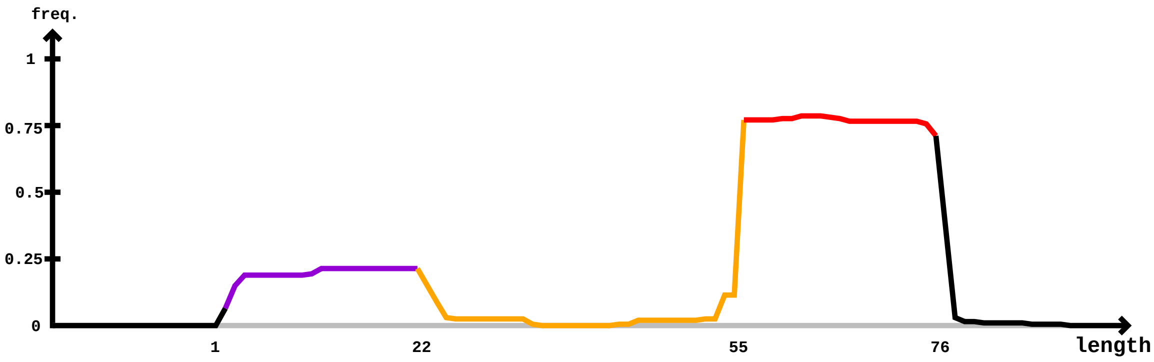


```
                   Star                                                        Mature
5'- ggugcucacugcagagccauuugggcagagcugagcuccagugaggggcucaugcucccccuggcuggagcgcagcucugcucaaauggcucugcagcgagcagugucuc  -3'  obs
    ggugcucacugcagagccauuugggcagagcugagcuccagugaggggcucaugcucccccuggcuggagcgcagcucugcucaaauggcucugcagcgagcagugucuc       exp
    (.(((((.(((((((((((((((((((((((((.((((((((.(((((.........))))).)))))))).))))))))))))))))))))))))).))))).).....  reads   mm   sample
    ................ccauuugggcagagcugagcu.........................................................................  3       0    Gu2
    .................cauuugggcagagcugagcu.........................................................................  1       0    Gu2
    .................cauuugggcagagcugagcuc........................................................................  1       0    Gu2
    .................cauuugggcagagcugagcucU.......................................................................  1       1    Gu2
    .................cauuugggcGgagcugagcucc.......................................................................  1       1    Gu2
    ...........................................................ccuggcuggagcgcagcucug..............................  1       0    Gu2
    ...........................................................ccuggcuggagcgcagcucugc.............................  2       0    Gu2
    ....................................................................agcgcagcucugcucaaauggA....................  1       1    Gu2
    ....................................................................agcgcagcucugcucaaauggG....................  1       1    Gu2
    ....................................................................agcgcagcucugcucaaauggcu...................  1       0    Gu2
    ......................................................................cgcagcucugcucaaauggc....................  2       0    Gu2
    ......................................................................cgcagcucugcucaaauggcC...................  13      1    Gu2
    ......................................................................cgcagcucugcucaaauggcA...................  1       1    Gu2
    ......................................................................cgcagcucugcucaaauggcu...................  6       0    Gu2
    ......................................................................cgcagcucugcucaaauggcuA..................  1       1    Gu2

    .................cauuugggcagagcugagcuc........................................................................  1       0    Ov3
    ..................................................................ggGgcgcagcucugcucaaauggcu...................  1       1    Ov3
    ......................................................................cgcagcucugcucaaaugg.....................  1       0    Ov3
    ......................................................................cgcagcucugcucaaGuggcu...................  1       1    Ov3
    ......................................................................cgcagcucugcucaaauggcu...................  4       0    Ov3
    ......................................................................cgcagcucugcucaaauggcC...................  5       1    Ov3
    ......................................................................cgcagcucugcucaaauggcA...................  2       1    Ov3

    ................ccauuugggcagagcugagcu.........................................................................  4       0    Gu1
    .................cauuugggcagagcugagcu.........................................................................  1       0    Gu1
    .................cauuugggcagagcugagcuc........................................................................  5       0    Gu1
    .................cauuugggcagagcugagcucA.......................................................................  1       1    Gu1
    .................cauuugggcagagcugagcucc.......................................................................  1       0    Gu1
    .................cauuugggcagagcugagcucca......................................................................  1       0    Gu1
    ..................auuugggcagagcugagcuc........................................................................  1       0    Gu1
    ..................auuugggcagagcugagcucc.......................................................................  2       0    Gu1
    ..........................agagcugagcuccagugagggC..............................................................  1       1    Gu1
```

```
                Star                                             Mature
ggugcucacugcagagccauuugggcagagcugagcuccagugaggggcucaugcucccccuggcuggagcgcagcucugcucaaauggcucugcagcgagcagugucuc
..........................agagcugagcuccagugagggg..............................................................  1   0  Gu1
.........................................................ccccuggcuggagcgcagcucu...............................  1   0  Gu1
....................................................................agcgcagcucugcucaaaugg.....................  1   0  Gu1
....................................................................agcgcagcucugcucaaauggc....................  2   0  Gu1
....................................................................agcgcagcucugcucaaauggcu...................  1   0  Gu1
....................................................................Cgcgcagcucugcucaaauggcu...................  1   1  Gu1
....................................................................agcgcagcucugcucaaauggcC...................  7   1  Gu1
......................................................................cgcagcucugcucaaauggcG...................  1   1  Gu1
......................................................................cgcGgcucugcucaaauggcu...................  3   1  Gu1
......................................................................cgcagcucugcucaaauggcC...................  17  1  Gu1
......................................................................cgcagcucugcucaaauggcu...................  26  0  Gu1
......................................................................cgcagcucugcucaGauggcu...................  3   1  Gu1
......................................................................cgcagcucugcucaaauggcA...................  1   1  Gu1
......................................................................cgcagcucugcucaaauggcuc..................  1   0  Gu1
......................................................................cgcagcucugcucaaauggcCc..................  1   1  Gu1
......................................................................cgcagcucugcucaaauggcuU..................  5   1  Gu1
......................................................................cgcagcucugcucaaauggcCcu.................  1   1  Gu1
......................................................................cgcagcucugcucaaauggcucC.................  1   1  Gu1

.................cauuugggcagagcugagcucc.......................................................................  1   0  Ov1
..................auuugggcagagcugagcucc.......................................................................  1   0  Ov1
....................................................................agcgcagcucugcucaaauggcC...................  1   1  Ov1
......................................................................cgcagcucugcucaGauggc....................  1   1  Ov1
......................................................................cgcagcucugcucaaauggcu...................  2   0  Ov1
......................................................................cgcagcucugcucaaauggcC...................  6   1  Ov1
......................................................................cgcagcucugcucaaauggcCc..................  1   1  Ov1
......................................................................cgcagcucugcucaaauggcucC.................  1   1  Ov1
............................................................................ucugcucaaauggcucugcagcgagcaC......  1   1  Ov1

................ccauuugggcagagcugagcu.........................................................................  5   0  Gu3
................ccauuugggcagagcugagcuc........................................................................  1   0  Gu3
.................cauuugggcagagcugagcucc.......................................................................  1   0  Gu3
..................auuugggcagagcugagcuc........................................................................  1   0  Gu3
..................auuugggcagagcugagcucc.......................................................................  3   0  Gu3
.........................cagagcugagcuccagugagggU..............................................................  1   1  Gu3
..........................agagcugagcuccagugagggC..............................................................  1   1  Gu3
..........................agagcugagcuccagugaggggc.............................................................  1   0  Gu3
....................................................................agcgcagcucugcucaaauggc....................  1   0  Gu3
....................................................................agcgcagcucugcucaaauggA....................  1   1  Gu3
......................................................................cgcagcucugcucaaauggcA...................  2   1  Gu3
......................................................................cgcagcucugcucaaauggcu...................  7   0  Gu3
......................................................................cgcagcucugcucaGauggcu...................  1   1  Gu3
......................................................................cgcagcucugcucaaauggcC...................  6   1  Gu3
......................................................................cgcagcucugcucaaauggcuU..................  2   1  Gu3
......................................................................cgcagcucugcucaaauggcCc..................  1   1  Gu3

.................cauuugggcagagcugagcuc........................................................................  1   0  Ov2
......................................................................cgcagcucugcucaaauggcu...................  2   0  Ov2
......................................................................cgcagcucugcucaaauggcC...................  4   1  Ov2
..........................................................................gcucugcucaGauggcucugc...............  1   1  Ov2
............................................................................ucuCcucaaauggcucugcagcga..........  1   1  Ov2
```
